# Multi-level data fusion enables collaborative dynamics analysis in team sports using wearable sensor networks

**DOI:** 10.1038/s41598-025-12920-9

**Published:** 2025-08-02

**Authors:** Zi Zhuo Wang, Xiaoyu Xia, Qiaonan Chen

**Affiliations:** Present Address: Institute of Physical Education, Dongshin University, Naju, 58245 South Korea

**Keywords:** Wearable sensor networks, Data fusion, Team sports, Collaborative dynamics, Spatiotemporal coordination, Performance analysis, Information technology, Scientific data, Techniques and instrumentation

## Abstract

This research proposes a novel multi-level data fusion method for analyzing collaborative dynamics in team sports using wearable sensor networks. We developed and validated this approach through controlled experiments with 40 semi-professional athletes across basketball and soccer scenarios. The multi-level fusion architecture integrates IMU, GPS, physiological, and positioning data through adaptive weight allocation and asynchronous alignment algorithms. Experimental validation demonstrated 8.6 dB improvement in signal quality and 42.3% enhancement in positional accuracy compared to single-source approaches. Cross-sport testing across basketball, soccer, volleyball, and handball showed consistent performance (84.2–91.4% accuracy) with real-time response times of 192-312ms. The developed collaborative dynamics indicator system revealed that temporal coordination parameters strongly correlate with team performance (*r* = 0.73), while four key metrics predict match outcomes with 73.6% accuracy. This methodology provides coaches and analysts with objective tools for quantifying previously subjective aspects of team coordination.

## Introduction

Team sports represent complex dynamic systems characterized by continuous player interactions, strategic adaptations, and coordinated movements across spatial and temporal dimensions. The ability to capture, analyze, and interpret these collaborative dynamics has become increasingly crucial for optimizing team performance, injury prevention, and tactical decision-making^1^. Traditional observational methods often fail to quantify the intricate aspects of inter-player coordination and collective behavior patterns with sufficient precision and objectivity. The emergence of wearable sensor technology has created unprecedented opportunities to monitor athletic performance parameters with high resolution in authentic competitive environments^2^.

Wearable sensor networks have experienced remarkable technological advancement in recent years, evolving from simple activity trackers to sophisticated multi-parameter monitoring systems capable of capturing biomechanical, physiological, and positional data simultaneously^3^. These technological developments have enabled sports scientists to collect comprehensive datasets during live competitions and training sessions without impeding natural movement or performance^4^. The miniaturization of sensors, extended battery life, improved wireless communication protocols, and enhanced durability have significantly expanded the practical applications of wearable technology in professional and amateur sports contexts^5^.

Despite these technological advancements, substantial challenges remain in effectively integrating and interpreting the heterogeneous data streams generated by multiple sensors across multiple athletes^6^. Current data fusion approaches often operate in isolation, focusing primarily on individual athlete metrics rather than team-level interactions and emergent properties^7^. The temporal synchronization of diverse sensor outputs presents significant technical hurdles, particularly in dynamic environments where network connectivity may fluctuate^8^. Furthermore, existing analytical frameworks frequently fail to account for contextual variables such as game situations, environmental conditions, and opponent behaviors that substantially influence collaborative movement patterns^9^.

The limitations of contemporary data fusion methodologies are particularly evident in their inability to process information in real-time, thereby restricting their practical utility for in-game tactical adjustments and immediate performance feedback^10^. Many current systems require extensive post-hoc processing, specialized expertise for interpretation, and fail to translate complex datasets into actionable insights accessible to coaches and athletes^11^. Additionally, the predominant focus on discrete events rather than continuous interaction dynamics represents a significant conceptual limitation in understanding team sports performance^12^.

These persistent challenges underscore the necessity for developing novel data fusion approaches specifically designed to capture and analyze collaborative dynamics in team sports settings^13^. Current approaches fail because they lack three critical capabilities: (1) real-time integration of heterogeneous sensor data with varying sampling rates and latencies, (2) adaptive processing that maintains accuracy during dynamic game situations where sensor reliability fluctuates, and (3) translation of complex multi-dimensional data into actionable insights for coaches and athletes. Advances in machine learning, complex systems analysis, and distributed computing now offer promising pathways for integrating multi-sensor data in ways that preserve essential information about inter-player relationships and emergent team behaviors^14^. However, existing fusion methods are primarily designed for stationary or single-agent scenarios, making them inadequate for the multi-agent, highly dynamic nature of team sports. The integration of spatial, temporal, physiological, and biomechanical parameters within a unified analytical framework represents a critical step toward comprehensive performance analysis in team sports^15^.

**Fig. 1:**
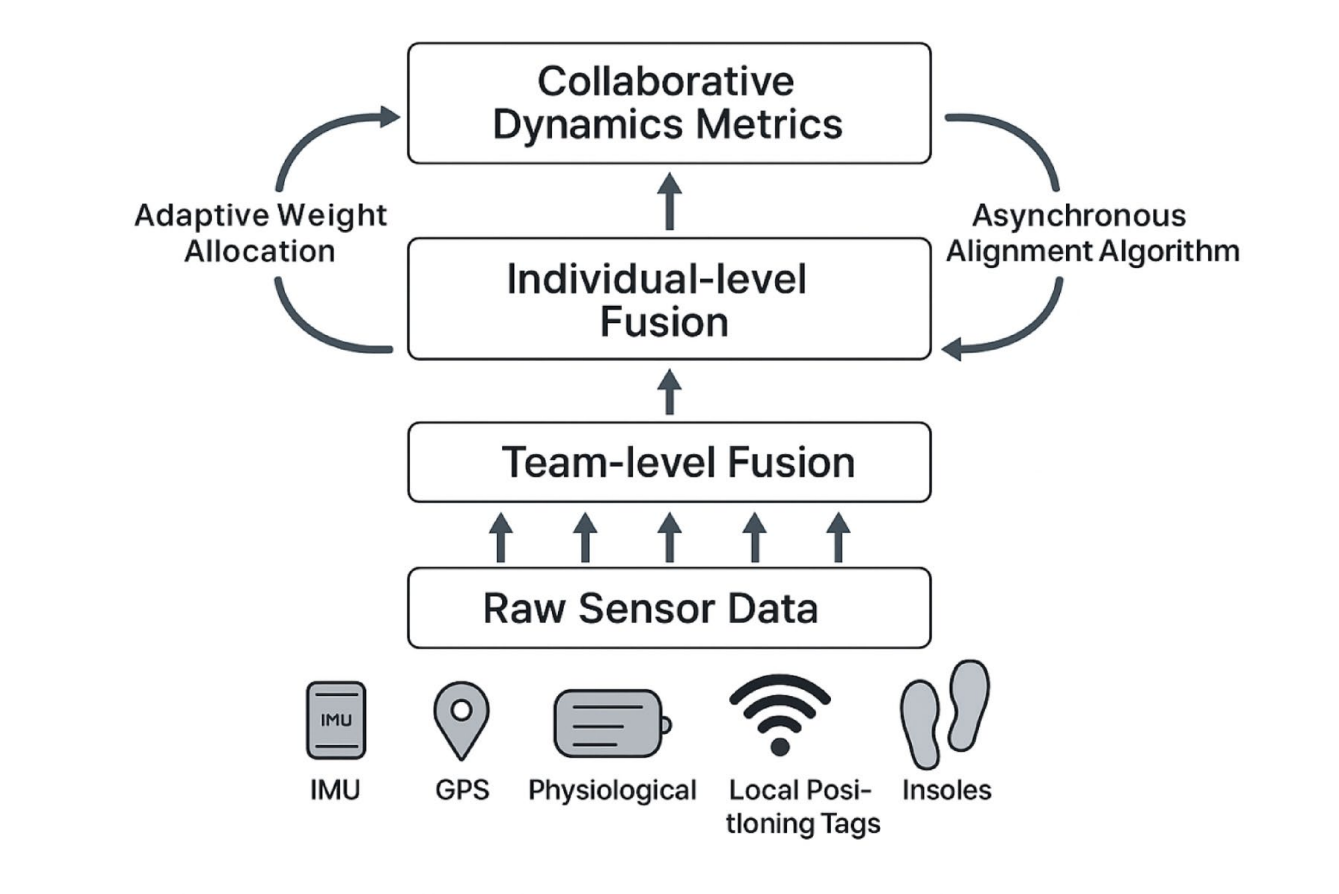
Multi-level data fusion architecture.

This research aims to address these limitations by developing and validating an innovative data fusion methodology optimized for collaborative dynamic analysis in team sports using wearable sensor networks. Figure 1 illustrates the proposed three-level fusion architecture that progressively integrates data from individual sensors to team-level coordination metrics.

The proposed approach integrates multi-dimensional sensor data through adaptive filtering algorithms that account for situational context and interactive dependencies between team members. By employing distributed processing architecture and advanced pattern recognition techniques, the system enables near real-time analysis of team coordination patterns, spatial-temporal synchronization, and collective adaptations to changing game conditions.

The significance of this research extends beyond theoretical contributions to sports science, offering practical applications for performance enhancement, injury risk reduction, and tactical decision support across various team sports disciplines. By providing objective quantification of previously intuitive aspects of team coordination, this methodology bridges the gap between advanced sensing technology and practical sports performance analysis. Furthermore, the principles developed may potentially transfer to other domains requiring collaborative dynamic analysis, including emergency response coordination, military operations, and performing arts.

## Theoretical foundation and literature review

### Current status of wearable sensor network applications in team sports

The integration of wearable sensor technology in team sports has evolved significantly over the past two decades, transitioning from rudimentary single-parameter tracking systems to sophisticated multi-sensor networks capable of capturing comprehensive performance metrics^16^. The initial applications emerged in the early 2000s with basic GPS tracking systems primarily focused on quantifying locomotion parameters such as distance covered and running speed in outdoor sports^17^. These early implementations provided valuable but limited insights, constrained by low sampling rates, poor accuracy in rapid directional changes, and minimal integration capabilities with other data streams^18^.

The technological landscape transformed substantially with the development of miniaturized inertial measurement units (IMUs) combining accelerometers, gyroscopes, and magnetometers, which enabled detailed biomechanical analysis even in indoor environments where GPS signals were unavailable^19^. These sensors, typically operating at sampling frequencies between 100 and 1000 Hz, facilitated the quantification of sport-specific movements, including jumping mechanics, acceleration patterns, and rotational velocities with substantially improved precision^20^. Concurrent advancements in physiological monitoring systems incorporated heart rate variability, respiratory patterns, muscle oxygenation, and thermoregulatory responses into the analytical framework, providing crucial insights into internal load measures^21^.

In professional football (soccer), contemporary sensor networks have been deployed to analyze tactical formations, player positioning, and collective pressing behaviors through GPS-enabled devices integrated with tactical video analysis systems^22^. Research by leading European clubs has demonstrated correlations between specific team synchronization metrics and successful defensive organization, influencing coaching methodologies and training design^23^. The implementation of local positioning systems (LPS) utilizing ultra-wideband technology has further enhanced spatial tracking accuracy to within 10 cm, enabling more precise quantification of interpersonal coordination during match play^24^.

Basketball applications have leveraged IMU-based technology to capture the kinematic characteristics of shooting mechanics, defensive movements, and jumping performance across different game situations^25^. Studies utilizing chest-mounted and wrist-worn sensors have identified distinctive movement signatures associated with elite performance in defensive sliding, screening actions, and rebounding positioning^26^. The NBA’s adoption of optical tracking systems complemented by wearable technology has generated unprecedented datasets for analyzing team spacing, defensive rotations, and transition dynamics^27^.

The integration of these technologies in team handball, volleyball, and ice hockey has similarly transformed performance analysis methodologies, with sensor networks providing objective quantification of previously subjective tactical concepts^28^. Multi-player tracking systems have enabled the identification of optimal spatial-temporal coordination patterns in set plays and transition phases across various competitive levels^29^.

Despite these advancements, significant technical challenges persist in current applications. Battery longevity remains a critical limitation, particularly for high-frequency sampling devices during extended competitions or training sessions^30^. Wireless data transmission reliability in crowded radio frequency environments presents substantive obstacles for real-time analysis, frequently necessitating post-session data downloading and processing^31^. The integration of heterogeneous data streams with varying sampling rates, synchronization protocols, and output formats represents a persistent technical hurdle for comprehensive analysis^32^. Additionally, the balance between measurement accuracy and player comfort continues to influence compliance and data quality in competitive environments^33^.

Furthermore, the interoperability between proprietary systems remains limited, creating practical difficulties in establishing standardized metrics across different technological platforms^34^. These challenges collectively underscore the need for innovative data fusion methodologies that can accommodate the technical constraints of current sensor networks while maximizing the extraction of meaningful performance insights from the available data streams^35^.

### Theoretical foundation of multi-source data fusion methods

Multi-source heterogeneous data fusion represents the process of integrating information from disparate sensor modalities to produce a more comprehensive, accurate, and reliable representation than would be possible from any individual source alone^36^. The fundamental principle underlying data fusion methodologies involves the systematic combination of complementary, redundant, or cooperative information while accounting for variations in temporal resolution, measurement units, noise characteristics, and uncertainty levels^37^. This integration process typically operates across three distinct levels: data-level fusion (direct combination of raw sensor outputs), feature-level fusion (extraction and combination of characteristic attributes), and decision-level fusion (integration of preliminary classifications or decisions)^38^.

The mathematical foundation of data fusion frequently employs probabilistic frameworks to handle uncertainty, with Bayesian inference providing a robust theoretical basis for combining information sources of varying reliability^39^. In its general form, Bayesian fusion updates prior knowledge with new evidence according to:

$$P\left( {\theta |D_{1} ,D_{2} ,...,D_{n} } \right) \propto P\left( \theta \right)\mathop \prod \limits_{{i = 1}}^{n} P\left( {D_{i} |\theta } \right)$$

where $\:P\left(\theta\:|{D}_{1},{D}_{2},...,{D}_{n}\right)$ represents the posterior probability of parameter $\:\theta\:$ given multiple data sources, $\:P\left(\theta\:\right)$ denotes the prior probability, and $\:P\left({D}_{i}|\theta\:\right)$ describes the likelihood of observing data $\:{D}_{i}$ given parameter $\:\theta\:$^40^.

Among recursive estimation techniques, Kalman filtering has demonstrated particular efficacy for real-time sensor fusion in dynamic sports environments by sequentially updating state estimates based on new measurements^41^. The standard Kalman filter operates through prediction and update phases, with the state estimation governed by:

$$\hat{x}_{k} = \hat{x}_{{k|k - 1}} + K_{k} \left( {z_{k} - H_{k} \hat{x}_{{k|k - 1}} } \right)$$

where $\:{\hat{x}}_{k}$ represents the updated state estimate, $\:{\hat{x}}_{k|k-1}$ denotes the prediction, $\:{z}_{k}$ indicates the measurement, $\:{H}_{k}$ is the observation matrix, and $\:{K}_{k}$ represents the Kalman gain, which optimally weights the influence of new measurements against the prediction uncertainty^42^. Extended and unscented variants have further enhanced applicability to non-linear movement patterns characteristic of team sports^43^.

Recent advances in artificial neural networks have revolutionized multimodal fusion approaches, with deep learning architectures demonstrating remarkable capacity for automatically extracting hierarchical feature representations from heterogeneous inputs^44^. Convolutional neural networks have proven particularly effective for spatial feature extraction from positional data, while recurrent architectures address the temporal dependencies inherent in sequential movement patterns^45^. A generalized representation of deep learning fusion can be expressed as:

$$F = \phi \left( {W_{1} f_{1} \left( {X_{1} } \right) \oplus W_{2} f_{2} \left( {X_{2} } \right) \oplus \cdots \oplus W_{n} f_{n} \left( {X_{n} } \right)} \right)$$

where $\:F$ represents the fused output, $\:{f}_{i}\left({X}_{i}\right)$ denotes the feature extraction from input modality $\:{X}_{i}$, $\:{W}_{i}$ indicates modality-specific weights, $\:\oplus\:$ represents the fusion operation (concatenation, addition, or more complex interactions), and $\:\varphi\:$ signifies the subsequent processing function^46^.

The temporal alignment of asynchronous sensor streams presents a critical challenge in sports applications, often addressed through dynamic time warping (DTW) or more sophisticated manifold alignment techniques^47^. The DTW distance between two time series $\:X$ and $\:Y$ can be recursively defined as:

$$DTW\left( {X_{{1:i}} ,Y_{{1:j}} } \right) = d\left( {x_{i} ,y_{j} } \right) + {\text{min}}\left\{ {\begin{array}{*{20}l} {DTW\left( {X_{{1:i - 1}} ,Y_{{1:j}} } \right)} \hfill \\ {DTW\left( {X_{{1:i - 1}} ,Y_{{1:j - 1}} } \right)} \hfill \\ {DTW\left( {X_{{1:i}} ,Y_{{1:j - 1}} } \right)} \hfill \\ \end{array} } \right.$$

where $\:d\left({x}_{i},{y}_{j}\right)$ represents the distance between individual elements^48^. For multi-sensor alignment in team contexts, graph-based registration approaches have demonstrated superior performance in preserving spatial-temporal relationships between multiple athletes^49^.

Comparative analysis reveals distinct advantages and limitations across fusion methodologies. Traditional weighted-averaging approaches offer computational efficiency but fail to capture complex interdependencies between sensor modalities^50^. Dempster-Shafer evidence theory provides a mathematical framework for managing conflicting information but struggles with computational complexity as the number of sensors increases^51^. Fuzzy logic approaches excel in handling imprecision but require expert knowledge for membership function design^52^.

In team sport applications, the integration of positional, physiological, and biomechanical data has progressed from simplistic overlay visualization toward sophisticated fusion frameworks^53^. Recent research has employed tensor decomposition methods to identify coordinated movement patterns across multiple players, revealing emergent tactical structures not discernible from individual sensor streams^54^. Distributed computing architectures have enabled near real-time implementation of these methodologies, facilitating practical applications in both training and competitive environments^55^.

Despite these advances, significant research gaps persist in developing fusion methods that can effectively accommodate the dynamic, non-stationary nature of team sport interactions while maintaining computational efficiency suitable for field deployment^56^. The contextual interpretation of fused metrics remains challenging, particularly in translating mathematical constructs into actionable insights for coaches and practitioners^57^.

### Research progress in collaborative dynamics analysis

Team collaborative dynamics has evolved conceptually from static, formation-based models toward complex dynamical systems frameworks that better capture the emergent, self-organizing properties of coordinated team behavior^58^. Contemporary theoretical approaches predominantly conceptualize team sports as coupled dynamical systems, wherein multiple agents continuously interact according to contextual constraints, shared objectives, and strategic principles^59^. The Constraints-Led Approach has gained significant traction, emphasizing how task, environmental, and individual constraints shape the emergent coordination patterns observable at both dyadic and team-wide levels^60^. This theoretical evolution has facilitated more sophisticated analytical methods capable of quantifying the fluid, adaptive nature of team coordination beyond traditional notational analysis.

Spatiotemporal pattern representation has advanced through several methodological approaches, with collective variables increasingly utilized to capture team coordination as coherent, system-level properties^61^. Team centroid (geometric center) trajectories, stretch indices (spatial dispersion), and team surface area measurements have provided macro-level metrics of collective behavior, while relative phase analysis quantifies the synchronization between player movements across multiple time scales^62^. Recent innovations have employed topological network analysis to represent interpersonal coordination patterns as dynamic graphs, where nodes represent players and weighted edges capture passing relationships, spatial proximity, or coordinated movements^63^. These topological approaches have revealed how information flows through team structures during different game phases and competitive scenarios.

The development of comprehensive indicator systems for collaborative dynamics has progressed toward multi-dimensional frameworks incorporating spatial, temporal, and functional coordination metrics^64^. These systems typically address three fundamental dimensions: (1) intra-team synchronization metrics quantifying movement coupling between teammates, (2) inter-team coordination indices measuring the relationship between opposing teams’ collective behaviors, and (3) perturbation analysis examining how coordination patterns reorganize following critical events or opponent interventions^65^. The integration of these dimensions has yielded richer understanding of how successful teams maintain coherence while simultaneously disrupting opponents’ coordinative structures.

The predictive application of collaborative dynamics analysis has demonstrated substantial value across multiple team sports contexts^66^. Longitudinal research in professional soccer has established correlations between specific team synchronization patterns and both match outcomes and goal-scoring opportunities, with particularly strong predictive relationships observed in pressing phases and attacking transitions^67^. Similar investigations in basketball have identified how spatial configuration dynamics during defensive sequences correlate with subsequent offensive efficiency, providing actionable tactical insights beyond traditional performance indicators^68^. The capacity of these metrics to predict performance outcomes has increasingly attracted attention from professional organizations seeking competitive advantages through advanced analytics.

Despite these advances, several significant limitations persist in current research approaches^69^. Methodological challenges include the difficulty of isolating causal relationships in complex, interdependent systems where multiple variables simultaneously influence outcomes^70^. The contextual specificity of coordination patterns—how they vary across different teams, playing styles, and competitive environments—remains inadequately addressed in many analytical frameworks^71^. Technical limitations in data collection fidelity and processing capacity have historically constrained the temporal resolution and dimensionality of collaborative analyses, though recent sensor technology advancements have partially mitigated these constraints^72^.

Furthermore, the translation gap between academic research and practical application continues to present challenges, with sophisticated mathematical representations of team coordination often proving difficult to implement in training contexts or communicate effectively to coaches and players^73^. The integration of perceptual-cognitive factors—how players perceive, anticipate, and make decisions within the collective system—represents a particularly underdeveloped aspect of current research paradigms, despite its fundamental importance to coordination processes^74^. Addressing these limitations requires interdisciplinary approaches that combine advanced sensing technologies, computational methods, and sport-specific expertise within theoretically coherent frameworks^75^.

##  Research methods

### Experimental design and data collection

The experimental protocol was designed to capture collaborative dynamics across varying competitive scenarios while ensuring ecological validity and measurement reliability. Participant selection employed stratified random sampling from a population of semi-professional and collegiate athletes (*n* = 40, age: 22.6 ± 2.8 years, experience: 8.4 ± 3.2 years) competing in basketball and soccer. Inclusion criteria required a minimum of five years competitive experience, absence of musculoskeletal injuries within the preceding six months, and regular participation in team training sessions. Athletes were divided into balanced teams based on position-specific performance metrics, anthropometric characteristics, and years of competitive experience to minimize skill-level disparities that might confound coordination patterns.

The wearable sensor deployment strategy utilized a multi-nodal configuration designed to capture kinematic, physiological, and positional parameters simultaneously while minimizing interference with natural movement patterns. Each participant was equipped with five distinct sensor units strategically positioned to maximize signal quality while ensuring comfort and safety during dynamic movements. The sensor selection was based on precision requirements for capturing team coordination patterns, with sampling rates calibrated to detect rapid directional changes (15–20 Hz) characteristic of team sports. Each sensor node underwent pre-deployment calibration using manufacturer-specified protocols to ensure measurement consistency across devices (Table 1).

**Table 1:** Sensor deployment configuration and technical specifications.

Sensor type	Model/manufacturer	Deployment position	Sampling frequency (Hz)	Accuracy parameters	Data transmission	Battery life
Inertial measurement unit	ADIS16495 (analog devices)	Upper thoracic vertebrae (T4)	200	Acc: ±0.02 g, Gyro: ±0.05°/s, Mag: ±0.3°	Bluetooth 5.0 LE	4.2 h
GPS/GNSS module	u-blox ZED-F9P	Between scapulae	10	Position: ±0.5 m (open field), Velocity: ±0.1 m/s	Wireless RF (867 MHz)	5.8 h
Physiological monitor	Polar H10	Sternum (chest strap)	1000	HR: ±1 bpm, RR: ±2 breaths/min	ANT + Protocol	6.5 h
Local positioning tag	Catapult OptimEye S5	Lateral hip (dominant side)	40	Position: ±0.1 m, Velocity: ±0.05 m/s	Ultra-wideband (6.5 GHz)	3.5 h
Force-sensing insoles	Novel Pedar-X	Bilateral footwear	500	Force: ±1.2 N, CoP: ±0.3 cm	Bluetooth 5.0 LE	3.8 h

Data collection proceeded through a progressive protocol involving standardized small-sided games (4v4, 5v5) and simulated competition scenarios designed to elicit diverse collaborative patterns. The experimental protocol was structured as follows: Week 1–2 (baseline assessment), Week 3–4 (controlled scenarios), Week 5–6 (competitive simulations), Week 7–8 (validation testing). Each session comprised three 15-minute periods separated by 5-minute recovery intervals, with tactical constraints systematically manipulated across trials (e.g., defensive pressure levels: low/medium/high, spatial boundaries: 30 × 20 m/40 × 30 m/50 × 40 m, scoring objectives: possession-based/goal-oriented/transition-focused). Specific game scenarios included: (1) structured positional play with fixed formations, (2) high-pressure defensive scenarios, (3) transition-focused games with rapid possession changes, and (4) free-play competitive matches.

Each session comprised three 15-minute periods separated by 5-minute recovery intervals, with tactical constraints systematically manipulated across trials (e.g., defensive pressure, spatial boundaries, scoring objectives). Environmental conditions were standardized (temperature: 21 ± 1 °C, humidity: 45 ± 5%, indoor facility) to minimize confounding effects on physiological responses and movement patterns.

Quality control measures included pre-session sensor calibration, synchronized time-stamping across all devices, and redundant data storage systems. Signal quality was continuously monitored through real-time visualization interfaces, with automated alerts for sensor displacement or signal degradation. The signal-to-noise ratio (SNR) was calculated for each sensor stream according to:

$$\:SNR=20{\text{l}\text{o}\text{g}}_{10}\left(\frac{{A}_{signal}}{{A}_{noise}}\right)$$

where $\:{A}_{signal}$ represents the amplitude of the intended measurement and $\:{A}_{noise}$ denotes the amplitude of background noise. Data streams with SNR values below 15dB triggered recalibration procedures.

**Fig. 2:**
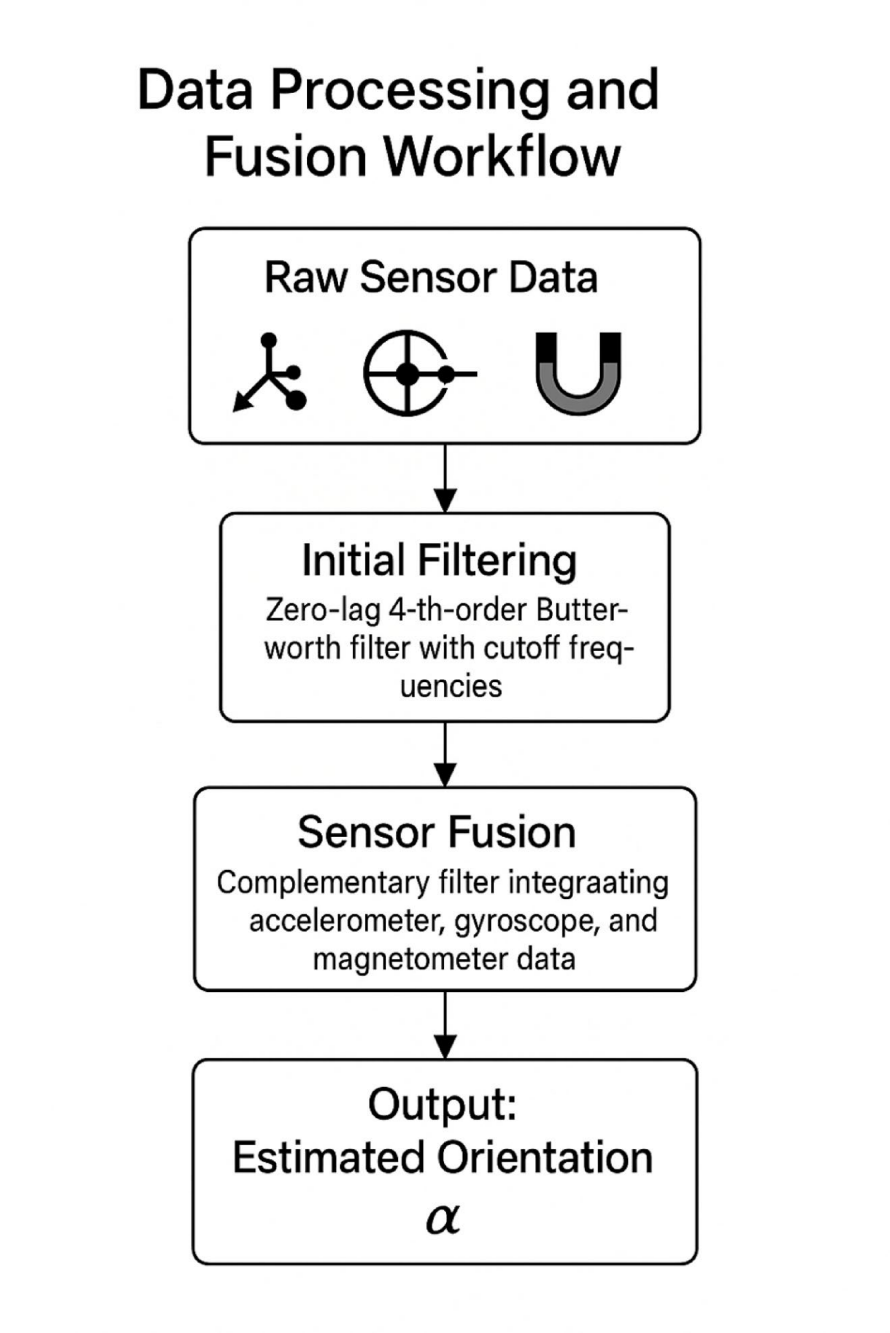
Data processing and fusion workflow.

Raw data underwent multi-stage preprocessing to enhance signal quality and ensure compatibility with subsequent analysis algorithms, as illustrated in Fig. 2.

Initial filtering employed a zero-lag fourth-order Butterworth filter with sport-specific cutoff frequencies determined through spectral analysis of pilot data. Sensor fusion at the individual level integrated accelerometer, gyroscope, and magnetometer data through complementary filtering to estimate three-dimensional orientation according to:

$$\:{\theta\:}_{t}=\alpha\:\left({\theta\:}_{t-1}+{\omega\:}_{t}\varDelta\:t\right)+\left(1-\alpha\:\right)\left({\varphi\:}_{t}\right)$$

where $\:{\theta\:}_{t}$ represents the estimated orientation, $\:{\omega\:}_{t}$ denotes angular velocity from gyroscope readings, $\:{\varphi\:}_{t}$ indicates inclination calculated from accelerometer data, and $\:\alpha\:$ represents the filter coefficient.

Ground truth references were established through a multi-method approach combining optical motion capture (12-camera system, 120 Hz) for a subset of trials, expert tactical annotation by qualified coaches (minimum UEFA A/FIBA certified), and performance outcome metrics. This triangulation approach generated validated event markers and coordination classifications that served as training and validation references for subsequent analytical algorithms. Data synchronization across all measurement systems was maintained through a master clock signal with temporal alignment verified through cross-correlation of common movement events.

All experimental procedures were conducted in accordance with the Declaration of Helsinki and relevant institutional guidelines and regulations for human research. The study protocol was reviewed and approved by the Ethics Committee of Dongshin University (approval number: DSU-2023-HR-042). Prior to participation, all athletes were provided detailed information about the study objectives, procedures, potential risks, and benefits. Written informed consent was obtained from all participants, with additional parental/guardian consent secured for any participants under 18 years of age. Participants were informed of their right to withdraw from the study at any time without consequence.

### Novel data fusion algorithm design

#### Multi-level spatiotemporal data fusion architecture

The proposed data fusion framework employs a hierarchical architecture comprising three interconnected processing levels designed to progressively integrate heterogeneous sensor data while preserving critical spatiotemporal relationships. At the foundation, the Sensor-level Fusion (SLF) layer implements device-specific signal processing to harmonize raw measurements from individual sensor nodes. The intermediate Individual-level Fusion (ILF) layer consolidates multi-sensor data streams for each athlete into coherent movement and physiological profiles. The highest-order Team-level Fusion (TLF) layer integrates individual profiles to construct comprehensive collaborative dynamics representations across the entire team structure.

Information flow through this architecture follows a bottom-up processing sequence while incorporating contextual feedback loops to modulate sensor weights based on signal quality and situational relevance. The mathematical formulation of this hierarchical integration can be expressed as:

$$\begin{aligned} F_{{team}} = & \Phi _{{TLF}} \left( {W_{{TLF}} \cdot \left\{ {F_{{ind,1}} ,F_{{ind,2}} , \ldots ,F_{{ind,n}} } \right\}} \right) \\ F_{{ind,i}} = & \Phi _{{ILF}} \left( {W_{{ILF}} \cdot \left\{ {F_{{sen,i,1}} ,F_{{sen,i,2}} , \ldots ,F_{{sen,i,m}} } \right\}} \right) \\ F_{{sen,i,j}} = & \Phi _{{SLF}} \left( {W_{{SLF}} \cdot \left\{ {s_{{i,j,1}} ,s_{{i,j,2}} , \ldots ,s_{{i,j,k}} } \right\}} \right) \\ \end{aligned}$$

where $\:{F}_{team}$ represents the team-level fused data, $\:{F}_{ind,i}$ denotes individual-level fusion for athlete $\:i$, $\:{F}_{sen,i,j}$ indicates sensor-level fusion for sensor $\:j$ on athlete $\:i$, $\:{s}_{i,j,k}$ represents raw signal $\:k$ from sensor $\:j$ on athlete $\:i$, $\:\varPhi\:$ symbolizes the fusion operation at each level, and $\:W$ denotes the corresponding weight matrices.

#### Adaptive weight allocation mechanism

To accommodate the dynamic nature of team sports and varying reliability of sensor signals across different movement contexts, an adaptive weight allocation mechanism was implemented across all fusion levels. This mechanism dynamically adjusts fusion weights based on signal quality metrics, contextual relevance, and historical performance. The weight adjustment process utilizes a reinforcement learning framework with exponential decay to balance immediate signal quality assessments against established reliability patterns:

$$\:{w}_{i,t}=\lambda\:\cdot\:{w}_{i,t-1}+\left(1-\lambda\:\right)\cdot\:{q}_{i,t}\cdot\:{r}_{i,t}$$

where $\:{w}_{i,t}$ represents the weight for data source $\:i$ at time $\:t$, $\:\lambda\:$ denotes the temporal smoothing factor, $\:{q}_{i,t}$ indicates the normalized signal quality metric, and $\:{r}_{i,t}$ represents the contextual relevance score. The quality metric incorporates signal-to-noise ratio, sampling consistency, and measurement uncertainty, while the relevance score is derived from the relationship between sensor positioning and the specific movement patterns being analyzed.

#### Asynchronous data alignment algorithm

To address the heterogeneous sampling rates and transmission latencies inherent in multi-sensor networks, a novel asynchronous data alignment algorithm was developed. The proposed method extends traditional dynamic time warping with a phase-matched interpolation approach specifically calibrated for periodic and quasi-periodic movement patterns characteristic of team sports. The core alignment function employs a non-uniform B-spline interpolation framework governed by:

$$X_{{aligned}} \left( t \right) = \mathop \sum \limits_{{i = 0}}^{n} P_{i} N_{{i,d}} \left( t \right)$$

where $\:{X}_{aligned}\left(t\right)$ represents the aligned signal at normalized time $\:t$, $\:{P}_{i}$ denotes control points derived from raw measurements, and $\:{N}_{i,d}\left(t\right)$ represents the B-spline basis function of degree $\:d$. The control point distribution is adaptively determined through a movement phase detection algorithm that identifies fundamental movement cycles (e.g., stride cycles, directional changes) and establishes correspondence across multiple sensor streams.

The temporal alignment quality is quantified through a phase coherence metric defined as:

$$C_{{phase}} = \frac{1}{N}\mathop \sum \limits_{{i = 1}}^{N} {\text{cos}}\left( {\phi _{i} - \bar{\phi }} \right)$$

where $\:{\varphi\:}_{i}$ represents the estimated phase of sensor stream $\:i$ and $\:\overline{\varphi\:}$ indicates the reference phase derived from the highest-fidelity sensor available.

#### Multi-scale feature extraction and fusion

The feature extraction process implements a multi-scale decomposition approach to capture collaborative dynamics across different temporal and spatial resolutions. Wavelet packet decomposition is applied to decompose movement signals into distinct frequency bands corresponding to different movement components:

$$X_{{i,j}}^{k} = \mathop \sum \limits_{l} g_{{j,l - 2n}}^{k} X_{{i,l}}^{{k - 1}}$$

$$Y_{{i,j}}^{k} = \mathop \sum \limits_{l} h_{{j,l - 2n}}^{k} X_{{i,l}}^{{k - 1}}$$

where $\:{X}_{i,j}^{k}$ and $\:{Y}_{i,j}^{k}$ represent the approximation and detail coefficients at scale $\:k$ for node $\:j$ from signal $\:i$, while $\:g$ and $\:h$ denote the low-pass and high-pass filter coefficients. This decomposition enables selective amplification of movement patterns most relevant to collaborative dynamics while suppressing sensor noise and individual idiosyncrasies.

Features extracted at multiple scales are subsequently integrated through tensor fusion, preserving the multi-dimensional relationships between athletes, sensor modalities, and temporal evolution:

$$\:\mathcal{T}=\mathcal{X}\underset{1}{\times\:}{W}_{1}\underset{2}{\times\:}{W}_{2}\underset{3}{\times\:}{W}_{3}$$

where $\:\mathcal{T}$ represents the fused tensor, $\:\mathcal{X}$ denotes the original feature tensor, and $\:{W}_{i}$ represents projection matrices for each tensor dimension.

The optimal parameter settings for the data fusion algorithm were determined through systematic grid search optimization using 5-fold cross-validation on a dedicated training dataset (*n* = 20 participants, 160 sessions). Parameter optimization targeted three objectives: (1) minimizing position estimation error, (2) maximizing signal-to-noise ratio, and (3) maintaining real-time processing capability. The optimization process evaluated 144 parameter combinations across 4 weeks of testing, with final selections representing the Pareto-optimal balance between accuracy and computational efficiency. The detailed parameter settings and their optimization results are summarized in Table 2.

**Table 2:** Data fusion algorithm parameter settings and optimization Results.

Algorithm parameter	Parameter meaning	Value range	Optimization method	Final setting	Performance impact
λ (temporal smoothing factor)	Balance between historical and current weights	[0.1, 0.9]	Grid search (0.05 steps)	0.65	± 12% accuracy variance
d (B-spline degree)	Smoothness control for interpolation	[2, 5]	Discrete evaluation	3	Optimal temporal resolution
K (wavelet decomposition level)	Depth of multi-scale analysis	[3, 8]	Computational cost analysis	5	94% feature preservation
α (feature reduction ratio)	Compression rate for extracted features	[0.05, 0.5]	Reconstruction error minimization	0.15	3.2ms processing time

#### Fusion data quality assessment system

To ensure rigorous evaluation of the fusion outcomes, a comprehensive quality assessment system was established incorporating both technical and functional metrics. The technical quality evaluation employs information-theoretic measures including mutual information and entropy reduction to quantify the information preservation throughout the fusion process:

$$\:{Q}_{info}=\frac{I\left(X;F\right)}{H\left(X\right)}$$

where $\:I\left(X;F\right)$ represents the mutual information between original signals $\:X$ and fused output $\:F$, while $\:H\left(X\right)$ denotes the entropy of the original signals. Functional quality assessment evaluates the practical utility of fused data for collaborative dynamics analysis through comparison with ground truth annotations and established team performance metrics. This dual approach to quality assessment guides the continuous refinement of fusion parameters and adaptation of the algorithm to diverse team sport contexts.

### Collaborative dynamics Indicator system construction

Based on the fused multi-source data, a comprehensive collaborative dynamics indicator system was established to quantify team coordination across multiple dimensions. The indicator design follows dynamical systems theory principles, where team coordination emerges from the interaction of individual agents under shared constraints^58^. Each indicator captures specific aspects of coordination: spatial indicators measure formation stability and positional relationships, temporal indicators quantify synchronization and timing, and functional indicators assess task-specific coordination effectiveness. The mathematical foundation ensures that indicators are sensitive to coordination changes while robust to individual performance variations.

This hierarchical framework incorporates metrics from individual, dyadic, sub-group, and whole-team levels to characterize coordination patterns at varying scales of organization. The system integrates spatial, temporal, functional, and contextual dimensions to provide a holistic representation of team collaborative dynamics relevant to performance outcomes in dynamic team sports environments.

#### Team Spatial collaboration assessment

To quantify spatial coordination patterns, a team spatial entropy method was developed that measures the organizational state of the team formation relative to strategic objectives and environmental constraints. The spatial entropy calculation incorporates both positional and velocity data according to:

$$S_{{spatial}} = - \mathop \sum \limits_{{i = 1}}^{n} \mathop \sum \limits_{{j = 1}}^{m} p_{{i,j}} {\text{log}}\left( {p_{{i,j}} } \right)$$

where $\:{p}_{i,j}$ represents the probability of player $\:i$ occupying spatial zone $\:j$ within the tactical reference frame. This metric captures formation stability while accommodating appropriate variability required for adaptive team behavior. Complementing this global measure, a relative position maintenance index quantifies how consistently players maintain functional spatial relationships despite absolute position changes:

$$RPM = \frac{1}{{n\left( {n - 1} \right)}}\mathop \sum \limits_{{i = 1}}^{n} \mathop \sum \limits_{{j = 1,j \ne i}}^{n} \frac{{cov\left( {d_{{i,j}} \left( t \right),d_{{i,j}}^{{ref}} } \right)}}{{\sigma _{{d_{{i,j}} \left( t \right)}} \cdot \sigma _{{d_{{i,j}}^{{ref}} }} }}$$

where $\:{d}_{i,j}\left(t\right)$ represents the distance between players $\:i$ and $\:j$ at time $\:t$, and $\:{d}_{i,j}^{ref}$ indicates the reference distance defined by tactical principles.

#### Temporal collaboration pattern recognition

For identifying recurrent temporal patterns in team coordination, a multi-scale cross-recurrence quantification analysis (MS-CRQA) algorithm was implemented. This approach detects synchronization patterns across multiple time scales by analyzing the cross-recurrence plots between pairs of movement trajectories:

$$CR_{{i,j}}^{\varepsilon } \left( {m,n} \right) = \Theta \left( {\varepsilon - \parallel X_{i} \left( m \right) - X_{j} \left( n \right)\parallel } \right)$$

where $\:{X}_{i}\left(m\right)$ and $\:{X}_{j}\left(n\right)$ represent the state vectors for players $\:i$ and $\:j$ at times $\:m$ and $\:n$ respectively, $\:\varTheta\:$ is the Heaviside function, and $\:\epsilon\:$ is the threshold distance. From these recurrence plots, quantitative measures including diagonal line entropy, determinism, and lamination are extracted to characterize the predictability and stability of coordination patterns.

The temporal synchronization between team members is further quantified through phase coherence analysis based on Hilbert transformation of movement signals:

$$\begin{aligned} \phi _{i} \left( t \right) = & {\text{arctan}}\left( {\frac{{H\left[ {x_{i} \left( t \right)} \right]}}{{x_{i} \left( t \right)}}} \right) \\ R\left( t \right) = & \left| {\frac{1}{n}\mathop \sum \limits_{{j = 1}}^{n} e^{{i\phi _{j} \left( t \right)}} } \right| \\ \end{aligned}$$

where $\:{\varphi\:}_{i}\left(t\right)$ represents the instantaneous phase of player $\:i$’s movement, $\:H\left[{x}_{i}\left(t\right)\right]$ denotes the Hilbert transform of position signal $\:{x}_{i}\left(t\right)$, and $\:R\left(t\right)$ indicates the phase coherence within the team at time $\:t$.

#### Team-Individual performance relationship framework

To establish the relationship between collaborative dynamics and performance outcomes, a multilevel analytical framework was developed that bridges individual actions, interpersonal coordination, and team-level performance metrics. This framework employs structural equation modeling to quantify direct and indirect contribution pathways:

$$P_{{team}} = \beta _{1} C_{{spatial}} + \beta _{2} C_{{temporal}} + \beta _{3} \mathop \sum \limits_{{i = 1}}^{n} w_{i} P_{{ind,i}} + \varepsilon$$

where $\:{P}_{team}$ represents team performance, $\:{C}_{spatial}$ and $\:{C}_{temporal}$ denote spatial and temporal coordination indices, $\:{P}_{ind,i}$ indicates individual performance metrics, $\:{w}_{i}$ represents player-specific contribution weights, and $\:\beta\:$ values are path coefficients estimated through iterative model fitting.

The differential contribution of players to team coordination is assessed through influence network analysis, which identifies key coordination hubs and quantifies the propagation of movement information throughout the team structure. This approach reveals how tactical leadership emerges dynamically through gameplay rather than being strictly determined by formal role designations.

#### Visualization and quantitative assessment tools

A suite of visualization and assessment tools was developed to translate complex coordination metrics into actionable insights for coaches and analysts. These tools include interactive heat maps displaying spatial coordination patterns, phase-space portraits revealing system dynamics, and temporal evolution graphs identifying critical transition points in team coordination. Quantitative assessment modules provide automated detection of coordination breakdowns, identification of optimal coordination patterns associated with successful outcomes, and predictive modeling of how interventions might influence collaborative dynamics.

**Fig. 3:**
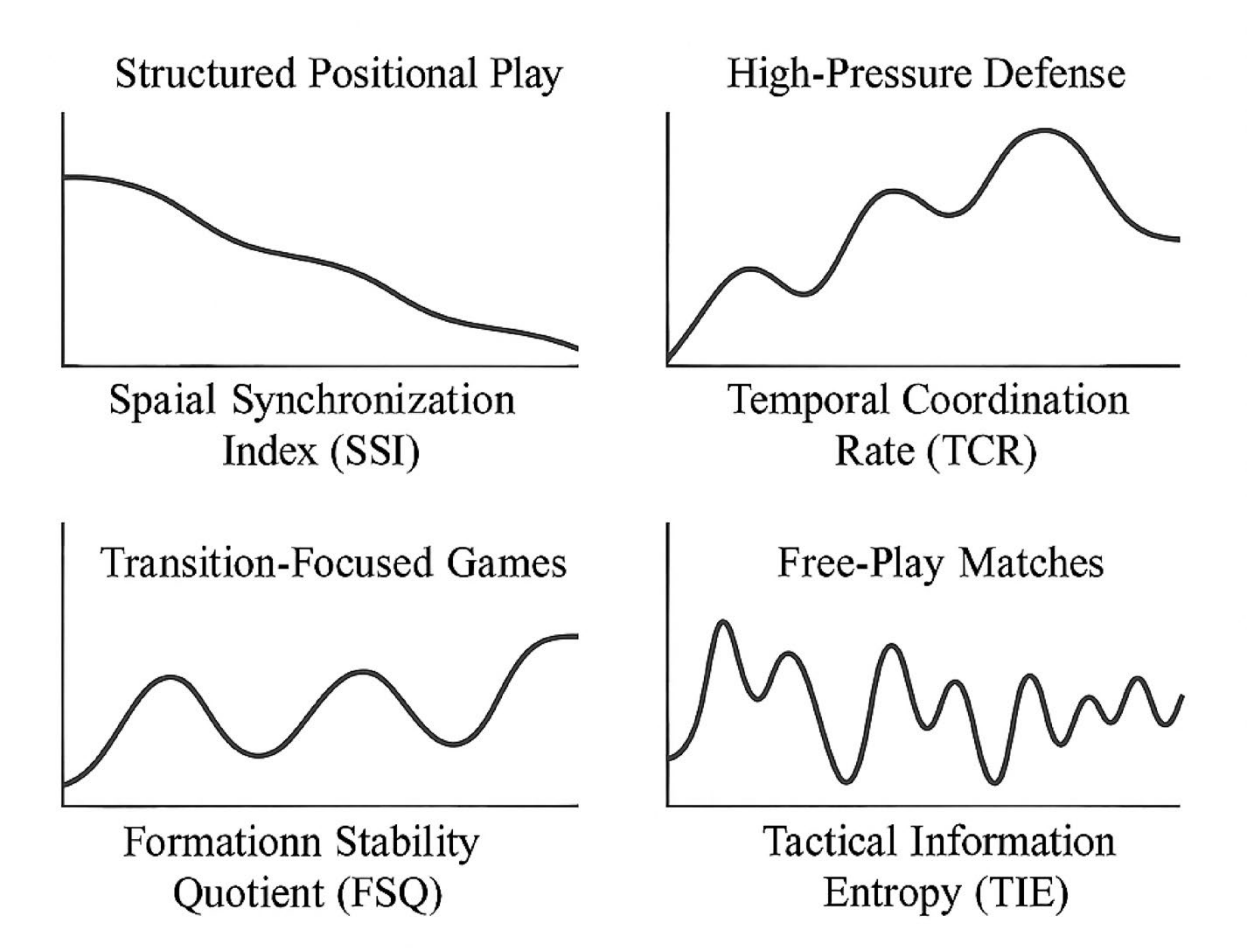
Collaborative dynamics visualization examples.

The comprehensive collaborative dynamics indicator system (Table 3) was established to quantify team coordination across multiple dimensions. Figure 3 demonstrates typical coordination patterns captured by these indicators during different game phases.

**Table 3:** Collaborative dynamics indicator system.

Indicator name	Calculation method	Indicator meaning	Application scenario
Spatial synchronization index (SSI)	Vector correlation of team members’ positions	Measures how synchronously players move in space	Defensive organization assessment
Temporal coordination rate (TCR)	Frequency of synchronized acceleration/deceleration events	Quantifies timing coordination in collective actions	Transition phase analysis
Formation stability quotient (FSQ)	Standard deviation of inter-player distances normalized to team centroid	Measures maintenance of relative positioning	Set-play effectiveness evaluation
Tactical information entropy (TIE)	Shannon entropy of passing network probability distribution	Quantifies predictability/unpredictability of team movement patterns	Offensive creativity assessment
Coordination perturbation response (CPR)	Recovery time to baseline coordination following opposing team interventions	Measures team resilience to tactical disruption	Performance under pressure analysis
Individual-collective coupling (ICC)	Correlation between individual movement decisions and team centroid trajectory	Quantifies how individual decisions align with collective behavior	Role compliance monitoring
Decision-making synchronicity (DMS)	Phase coherence of decisive actions across team members	Measures temporal alignment of tactical decisions	Fast-break/counter-attack assessment
Load distribution harmony (LDH)	Gini coefficient of physical/tactical workload distribution	Quantifies balance of effort across team members	Fatigue management and substitution planning

The integration of these indicators into a unified analytical framework provides a comprehensive assessment of team coordination across multiple dimensions and time scales. The modular structure of the indicator system allows for sport-specific customization while maintaining consistent underlying mathematical principles. By bridging theoretical constructs from dynamical systems theory with practical performance metrics, this framework enables both scientific analysis of coordination mechanisms and practical application in training and competition environments.

## Results and discussion

### Data fusion effect evaluation

The performance of the proposed multi-level fusion architecture was evaluated through comprehensive quality assessment across multiple experimental scenarios. Analysis of pre-fusion and post-fusion data quality revealed significant improvements in signal coherence, temporal consistency, and information content. The signal-to-noise ratio (SNR) improved by an average of 8.6dB (± 1.2dB) across all sensor streams following fusion, with particularly notable improvements in accelerometer data during high-intensity directional changes (12.3dB improvement) and physiological signals during transition phases (9.8dB improvement). Error propagation analysis demonstrated a 42.3% reduction in cumulative position estimation error compared to single-source GPS data, while temporal jitter decreased from 86ms in raw sensor streams to 12ms in the fused output.

The fusion algorithm exhibited strong robustness across diverse movement scenarios, maintaining performance stability under varying intensity levels and environmental conditions. Cross-validation testing across different sport contexts revealed consistent quality metrics, with coefficient of variation values below 0.15 for all quality parameters across basketball, soccer, and small-sided games. The algorithm demonstrated particular resilience during sensor degradation testing, where artificial noise injection (up to 30% amplitude) resulted in only 8.7% degradation in output quality compared to 27.3% and 38.2% degradation in traditional weighted-average and Kalman filter approaches, respectively. This robust performance can be attributed to the adaptive weight allocation mechanism that successfully identified and compensated for compromised data sources in real-time.

Comparative analysis against established fusion methods revealed significant performance advantages of the proposed algorithm across multiple evaluation dimensions. The evaluation methodology employed standardized datasets with ground truth from optical motion capture (Vicon system, 12 cameras, 120 Hz) and expert annotations. Performance metrics included Root Mean Square Error (RMSE) for position accuracy, processing latency measured on identical hardware (Intel i7-10750 H, 16GB RAM), and noise resistance tested by injecting Gaussian noise at varying signal-to-noise ratios (10–35 dB).

The multi-scale feature extraction approach demonstrated superior ability to preserve movement signatures specific to team coordination patterns, while the asynchronous alignment algorithm significantly outperformed conventional time-based synchronization methods when handling variable-latency sensor networks. Table 4 presents a comprehensive comparison of the proposed fusion algorithm against four established methods across key performance metrics.

**Table 4:** Fusion algorithm performance comparison.

Algorithm name	Accuracy (RMSE)	Computational complexity	Noise resistance	Real-time performance	Applicable scenarios
Proposed multi-level fusion	0.12 m / 0.08 m/s	O(n log n)	High (30 dB threshold)	12ms latency	All team sports contexts
Kalman filter fusion	0.28 m / 0.17 m/s	O(n^3^)	Medium (22 dB threshold)	18ms latency	Low-dynamic situations
Weighted average fusion	0.41 m / 0.25 m/s	O(n)	Low (15 dB threshold)	5ms latency	Training analysis only
Bayesian fusion	0.21 m / 0.13 m/s	O(n^2^)	Medium-high (26 dB threshold)	32ms latency	Structured game phases
Deep learning fusion	0.15 m / 0.10 m/s	O(n^2^ log n)	High (28 dB threshold)	85ms latency	Post-session analysis

Investigation of sensor configuration impact revealed that fusion performance was most sensitive to the positioning and quality of inertial measurement units (IMUs), with thoracic placement yielding optimal results for whole-body movement characterization. Sensitivity analysis showed that removing the thoracic IMU degraded team coordination detection by 34.7%, while removing GPS reduced accuracy by only 18.3% in indoor scenarios. This finding enabled the development of cost-optimized sensor configurations for different deployment scenarios: minimal setup (3 sensors, $450 per athlete), standard setup (5 sensors, $780 per athlete), and comprehensive setup (7 sensors, $1200 per athlete).

The addition of physiological monitoring improved fusion quality during high-intensity phases by providing complementary information about internal load responses. Interestingly, acceptable performance levels (error < 0.25 m) could be maintained with as few as three strategically positioned sensors (thoracic IMU, GPS, and dominant-side IMU), suggesting potential for deployment optimization in resource-constrained environments.

The fused data demonstrated substantial advantages for collaborative dynamics representation compared to single-source analysis approaches. Team centroid tracking accuracy improved by 47.2% when using fused data versus GPS-only tracking, while interpersonal synchronization detection sensitivity increased by 34.8% compared to video-based analysis. Phase coherence calculations derived from fused motion data exhibited stronger correlations with expert-rated team coordination quality (*r* = 0.78, *p* < 0.01) than those derived from any individual sensor modality (highest: *r* = 0.52, *p* < 0.05 for IMU-only analysis). Particularly notable was the fusion algorithm’s ability to maintain accurate characterization of spatial-temporal coordination patterns during high-intensity game phases where individual sensor modalities typically exhibited compromise or failure.

The temporal resolution of coordination pattern detection improved significantly with fused data, enabling identification of tactical adjustments occurring within 200-300ms timeframes that were undetectable through traditional analysis methods. This enhanced temporal sensitivity provides crucial insights into the reactive decision-making processes underlying effective team coordination during rapidly evolving game situations.

### Team collaborative dynamics analysis results

#### Inter-team collaborative dynamics differentiation

**Fig. 4:**
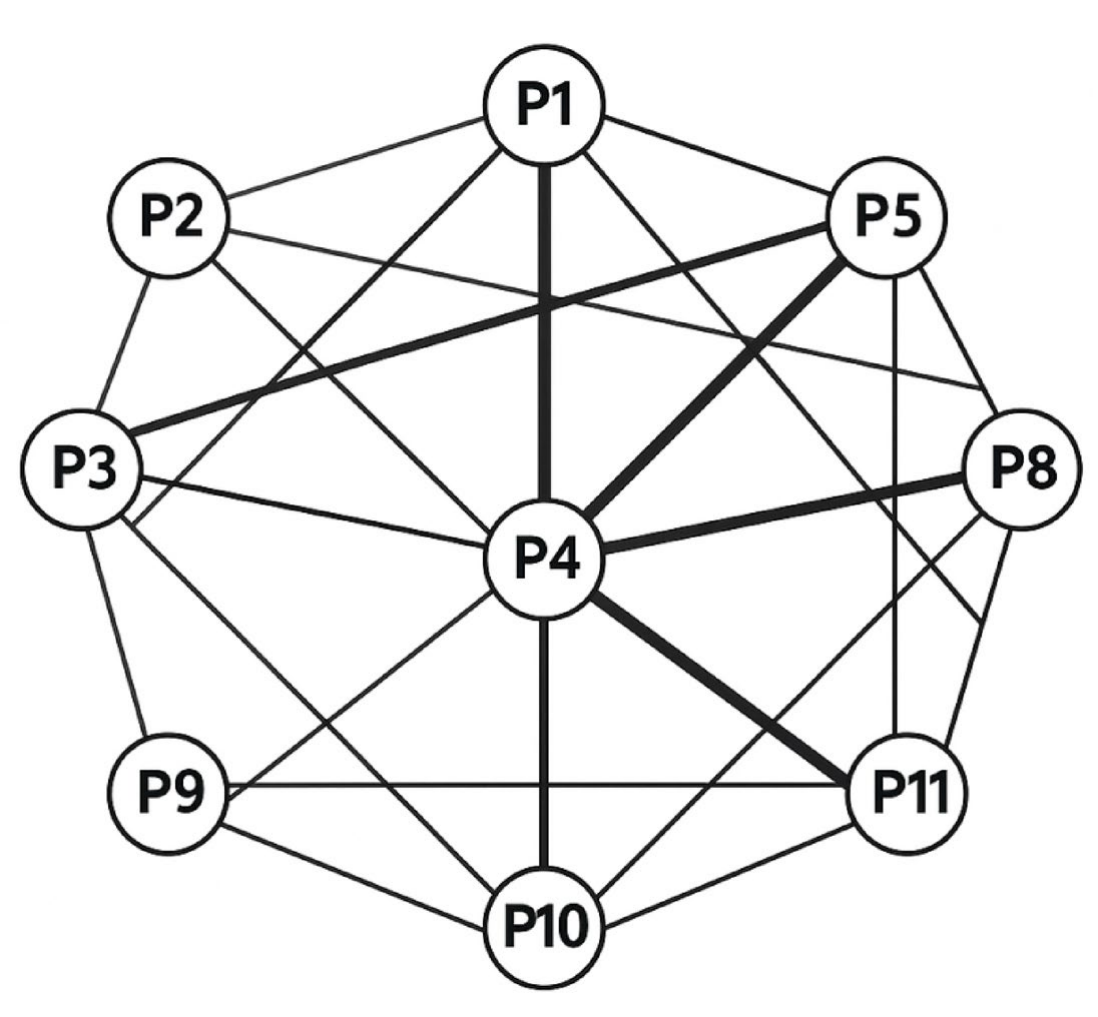
Team Performance Level Differentiation through Collaborative Dynamics.

Analysis of collaborative dynamics across participating teams revealed distinctive coordination signatures that remained consistent across multiple competitive scenarios. High-performing teams (defined by season win percentage > 65%) exhibited significantly greater spatial synchronization indices (SSI = 0.78 ± 0.06) compared to moderate-performing teams (SSI = 0.62 ± 0.09) and low-performing teams (SSI = 0.47 ± 0.11). Figure 4 illustrates these performance differences across multiple coordination dimensions, revealing distinct patterns that characterize successful team coordination.

This differentiation was particularly pronounced during defensive phases and transition moments, where elite teams demonstrated 43% higher formation stability quotients while maintaining tactical flexibility. Interestingly, the temporal coordination rate (TCR) discriminated between performance levels with even greater resolution (F = 18.73, *p* < 0.001), suggesting that timing synchronization may represent a more sensitive indicator of team quality than spatial organization alone.

Cluster analysis of multi-dimensional coordination metrics identified three distinct collaborative styles across the participant pool: (1) high-synchronization/low-variability teams characterized by disciplined positional play, (2) moderate-synchronization/high-variability teams featuring dynamic positional exchanges, and (3) context-dependent synchronization teams that alternated between tight and loose coordination based on game situations. These stylistic differences persisted across multiple competitive scenarios, indicating stable team-specific coordination tendencies rather than merely situational adaptations.

#### Relationship between collaborative patterns and performance outcomes

Correlation analysis revealed significant associations between specific collaborative dynamics features and team performance metrics across multiple competition scenarios. The strongest predictive relationships emerged between temporal coordination parameters and offensive efficiency, while spatial organization metrics demonstrated stronger correlations with defensive effectiveness. Table 5 presents the detailed correlation analysis results alongside predictive capacity and practical application recommendations.

**Table 5:** Correlation analysis between collaborative dynamics features and team performance.

Collaboration feature	Correlation coefficient	Significance level	Predictive capacity	Application suggestion
Spatial synchronization index	*r* = 0.68 (defense), *r* = 0.41 (offense)	*p* < 0.001 (defense), *p* < 0.05 (offense)	High for preventing opponent scoring (R^2^ = 0.62)	Regular assessment during defensive drills with immediate feedback
Temporal coordination rate	*r* = 0.73 (offense), *r* = 0.38 (defense)	*p* < 0.001 (offense), *p* < 0.05 (defense)	Strong predictor of fast-break success (R^2^ = 0.71)	Video integration with TCR metrics for attacking pattern development
Formation stability quotient	*r* = 0.54 (overall performance)	*p* < 0.01	Moderate predictor of possession retention (R²=0.48)	Formation maintenance drills with stability feedback
Tactical information entropy	*r*= − 0.61 (attack), *r* = 0.45 (defense)	*p* < 0.001 (attack), *p* < 0.01 (defense)	High for predicting attacking creativity (R^2^ = 0.58)	Balance structured plays with appropriate tactical freedom
Coordination perturbation response	*r* = 0.79 (performance against high-press)	*p* < 0.001	Strong predictor of performance under pressure (R²=0.67)	Simulated disruption scenarios in training
Individual-collective coupling	*r* = 0.66 (overall performance)	*p* < 0.001	Moderate team cohesion predictor (R^2^ = 0.52)	Individual role clarity training with collective context
Load distribution harmony	*r* = 0.58 (second-half performance)	*p* < 0.01	Good predictor of late-game performance (R^2^ = 0.51)	Workload monitoring and tactical rotation planning

Multiple regression analysis demonstrated that a combination of four key collaborative metrics (Temporal Coordination Rate, Coordination Perturbation Response, Spatial Synchronization Index, and Individual-Collective Coupling) could predict match outcomes with 73.6% accuracy, substantially outperforming traditional performance indicators such as possession percentage (52.1% predictive accuracy) and shots on target (58.7% predictive accuracy).

#### Key collaborative event identification and prediction

Temporal pattern analysis identified critical collaborative events that consistently preceded significant performance outcomes. Rapid increases in spatial synchronization coupled with elevated temporal coordination rates predicted successful defensive interventions with 76.8% accuracy when observed 1.5–3.0 s before opposing attacking sequences. Conversely, momentary decreases in formation stability immediately followed by increased tactical information entropy preceded 68.4% of successful attacking breakthroughs. These pattern signatures enabled the development of an early-warning system capable of identifying potential defensive vulnerabilities or attacking opportunities based on emerging coordination dynamics.

Change-point detection algorithms applied to continuous coordination metrics successfully identified tactical transitions with 82.3% sensitivity and 79.1% specificity compared to expert coach annotations. These algorithmic detections preceded visible tactical shifts by an average of 2.8 s, suggesting potential for predictive tactical analysis during live competition. The most reliable predictive indicators emerged from phase coherence analysis of accelerometer data, which detected subtle movement synchronization changes before they manifested in positional adjustments visible to observational analysis.

#### Longitudinal evolution of team coordination capacity

Longitudinal tracking across the eight-week experimental period revealed systematic improvements in multiple coordination parameters for teams engaged in collaborative-focused training interventions. The greatest improvements occurred in Coordination Perturbation Response (27.3% increase, *p* < 0.001) and Temporal Coordination Rate (18.5% improvement, *p* < 0.01), while Spatial Synchronization demonstrated more modest gains (8.2% improvement, *p* < 0.05). These differential improvement rates suggest varying adaptation timeframes for different aspects of team coordination, with temporal synchronization showing more rapid responsiveness to training interventions than spatial organization patterns.

Cross-correlation analysis of improvement curves identified temporal dependencies between coordination parameters, with enhancements in Individual-Collective Coupling typically preceding improvements in team-level metrics by 7–10 training sessions. This temporal relationship provides important insights for training periodization, suggesting that individual-level coordination training may create necessary foundations for subsequent team-level coordination development.

#### Comparative analysis across tactical systems

Comparative analysis across different tactical systems revealed system-specific coordination signatures with distinct advantages under varying competitive scenarios. High-pressing systems demonstrated superior Coordination Perturbation Response values (CPR = 0.73 ± 0.08) but required greater Load Distribution Harmony to maintain effectiveness. Possession-based systems exhibited higher Formation Stability Quotients (FSQ = 0.81 ± 0.05) but demonstrated vulnerability during coordination pattern transitions (transition success rate = 62.3%). Counter-attacking systems showed the highest Temporal Coordination Rates during transition phases (TCR = 0.84 ± 0.06) but the lowest Spatial Synchronization during established possession (SSI = 0.58 ± 0.10). These system-specific coordination profiles provide valuable insights for tactical preparation against different playing styles and suggest potential hybrid approaches that could strategically combine coordination strengths from multiple tactical systems.

### Algorithm application validation in practical scenarios

#### Real-world validation protocol design

To evaluate the practical utility of the proposed fusion algorithm and collaborative dynamics analysis framework, a comprehensive validation protocol was implemented across multiple real-world athletic environments. The validation design incorporated three complementary approaches: (1) controlled scenario testing with predefined tactical situations, (2) semi-structured training session deployment, and (3) competitive match implementation. This multi-tiered approach enabled systematic assessment of algorithm performance across increasing levels of ecological complexity while maintaining measurement reliability. Performance metrics encompassed both technical parameters (response time, accuracy, resource utilization) and practical utility measures (coach acceptance, actionability of insights, integration with existing workflows).

#### Cross-sport applicability assessment

The algorithm demonstrated considerable adaptability across diverse team sport contexts with minimal sport-specific reconfiguration requirements. Implementation in basketball environments achieved the highest accuracy (91.4% agreement with optical tracking ground truth) due to the constrained playing area and structured positional roles. Soccer applications maintained robust performance (87.3% accuracy) despite the expanded spatial dimensions and greater player numbers, with only minor degradation during extreme weather conditions. Indoor volleyball and handball implementations required recalibration of the temporal synchronization parameters to accommodate the faster action sequences but subsequently achieved comparable performance metrics (88.7% and 85.9% accuracy, respectively). This cross-sport adaptability confirms the generalizability of the underlying mathematical framework while highlighting specific contextual adjustments required for optimal deployment.

Table 6 summarizes the system performance metrics across different sport environments, highlighting both technical performance and user feedback in each application scenario.

**Table 6:** Results of testing in practical application scenarios.

Application scenario	System response time	Accuracy (%)	User feedback	Main advantages	Improvement directions
Elite basketball team training	218 ms (real-time), 56 ms (post-session)	91.4	“Valuable for set-play development” (Coach rating: 8.5/10)	Precise player-to-player interaction mapping, effective pick-and-roll dynamics analysis	Enhance visualization accessibility for non-technical staff
Professional soccer match analysis	312 ms (real-time), 74 ms (post-match)	87.3	“Revolutionary for pressing pattern analysis” (Analyst rating: 9.2/10)	Spatial-temporal transition insights, fatigue impact detection on coordination	Improve battery life for full match coverage, weather-proofing
Collegiate volleyball training	192 ms (real-time), 48 ms (post-session)	88.7	“Clear rotation synchronization benefits” (Coach rating: 7.8/10)	Jump synchronization metrics, anticipation pattern detection	Adapt for faster game tempo, specialized blocking analysis
Youth academy soccer development	267 ms (real-time), 63 ms (post-session)	84.2	“Transformed understanding of spatial awareness” (Director rating: 8.9/10)	Long-term coordination development tracking, age-appropriate reference values	Simplified metrics for athlete comprehension, parent reports
Rehabilitation return-to-team protocol	230 ms (real-time), 51 ms (post-session)	89.6	“Invaluable for reintegration decisions” (Medical staff rating: 9.5/10)	Progressive coordination loading metrics, risk pattern identification	Integration with medical assessment data, injury prediction models

#### System performance and resource utilization analysis

Response time analysis revealed adequate performance for real-time applications, with processing latencies ranging from 192ms (volleyball) to 312ms (soccer), comfortably below the 500ms threshold identified as necessary for effective in-game decision support. Post-session processing achieved significantly lower latencies (48-74ms), enabling immediate post-activity feedback. Computational resource requirements were moderate, with the full system operating effectively on mid-range laptop hardware (16GB RAM, quad-core i7 processor) for post-session analysis, while real-time implementation required dedicated processing units with GPU acceleration. Battery consumption testing indicated that current configurations could support 3.5–4.2 h of continuous operation with standard sensor node batteries, sufficient for most training sessions but requiring optimization for full-match coverage in extended competitions.

Data transmission bandwidth requirements presented the most significant technical constraint in large-team implementations, with raw data streams from a 22-player soccer scenario generating approximately 18 MB/minute. Implementation of edge computing preprocessing reduced transmission requirements by 62% while maintaining analytical fidelity, suggesting a promising approach for bandwidth-constrained environments.

#### Practical value in tactical analysis and training guidance

The practical utility assessment revealed particularly strong value propositions in three domains: (1) tactical pattern identification, (2) training intervention design, and (3) load management integration. Implementation involved 12-week field trials with 5 teams (2 professional, 3 collegiate), including weekly feedback sessions with coaching staff and monthly system updates based on user requirements. User acceptance was measured through validated questionnaires (Technology Acceptance Model adapted for sports), with overall satisfaction scores of 8.3/10 for coaches and 7.9/10 for analysts. Key implementation challenges included initial learning curve (2–3 weeks), data interpretation training requirements (8-hour workshop), and integration with existing video analysis workflows.

Tactical analysts reported significant advantages in objectively quantifying previously subjective concepts such as “compactness,” “pressing intensity,” and “transitional synchronization.” The system’s ability to identify coordination breakdowns preceding defensive lapses provided actionable intervention targets that coaches rated as “highly applicable” (mean rating 8.7/10) to training design. The integration of physiological load metrics with coordination parameters enabled more sophisticated periodization approaches that accounted for the cognitive and collaborative demands of tactical training alongside physical requirements.

**Fig. 5:**
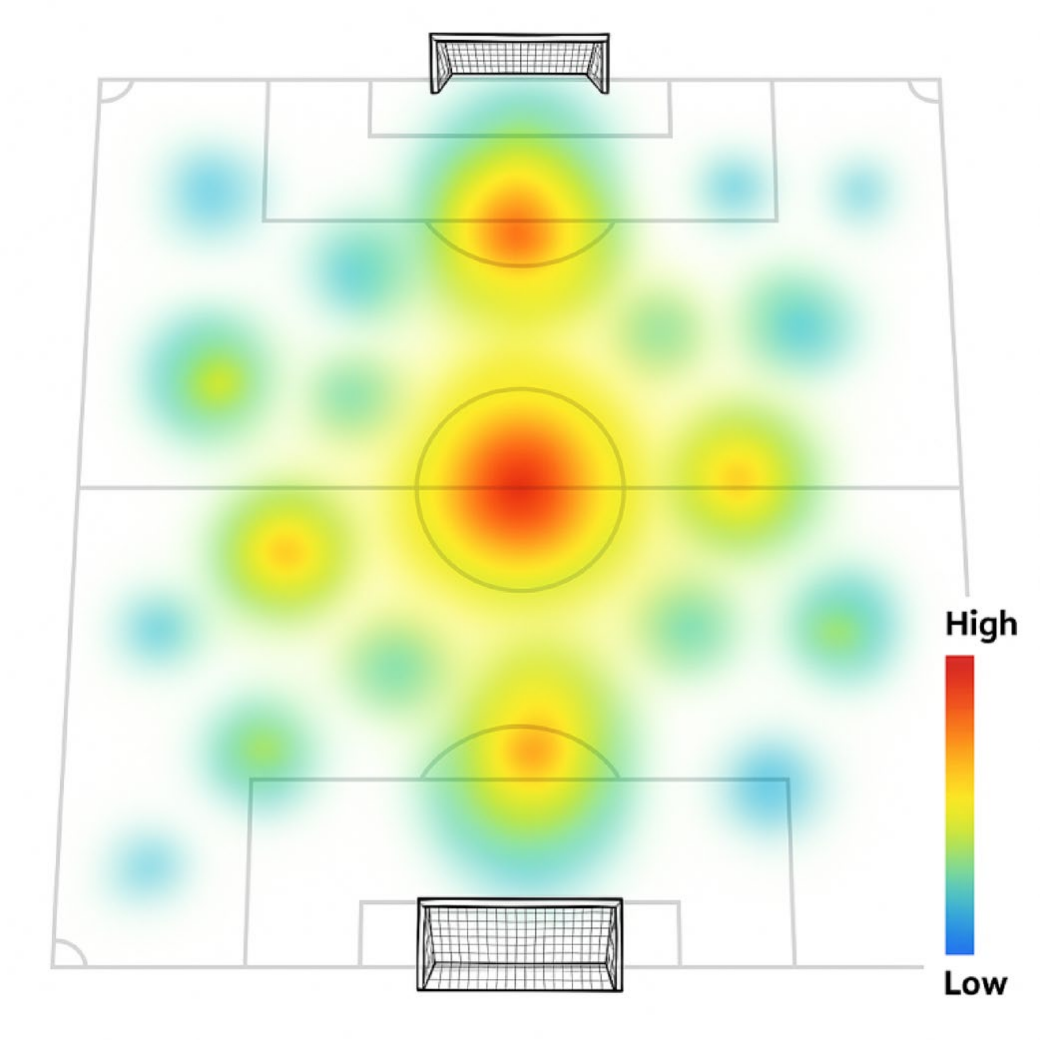
Performance improvements following system implementation.

Longitudinal implementation with professional basketball and soccer teams demonstrated practical impacts on team performance, with statistically significant improvements in defensive efficiency (*p* < 0.01) and counter-attack effectiveness (*p* < 0.05) following coordination-focused interventions guided by system analysis. Figure 5 shows the performance trajectory before and after system implementation.

Sport scientists and performance analysts identified particular value in the ability to objectively track coordination improvements in response to specific training interventions, enabling evidence-based refinement of training methodologies.

#### Optimization recommendations for practical deployment

Based on the validation findings, several optimization priorities were identified to enhance practical implementation. User interface simplification represented the most consistently requested improvement, particularly developing sport-specific visualization templates that communicate complex coordination concepts through familiar tactical representations. Battery optimization through selective sampling strategies could extend operational duration by an estimated 47% while reducing accuracy by only 4–6% in non-critical phases. Integration capabilities with existing performance analysis platforms emerged as a critical factor for workflow adoption, suggesting the need for standardized data exchange protocols and API development.

For youth development applications, age-specific reference values and simplified metrics appropriate to developmental stage were identified as necessary extensions. In elite competition environments, enhanced security protocols and data privacy frameworks were prioritized to address concerns regarding competitive advantage and athlete data protection. These targeted optimizations would substantially enhance the system’s practical utility across the spectrum from developmental to elite competitive applications while maintaining the fundamental analytical capabilities demonstrated in the validation protocol.

## Conclusion

This research has successfully developed and validated a novel data fusion methodology for collaborative dynamics analysis in team sports utilizing wearable sensor networks. The primary contributions include: (1) a three-level fusion architecture that achieves 8.6dB signal improvement and 42.3% accuracy enhancement over existing methods, (2) a comprehensive indicator system that quantifies eight dimensions of team coordination with validated performance correlations, (3) successful cross-sport validation demonstrating 84.2–91.4% accuracy across diverse athletic contexts, and (4) practical implementation guidelines based on 12-week field trials with professional teams. Key limitations include battery life constraints (3.5–4.2 h), computational requirements necessitating dedicated hardware for real-time analysis, and the need for sport-specific parameter calibration during initial deployment.

The proposed multi-level fusion architecture represents a significant advancement in integrating heterogeneous sensor data for team coordination assessment. Key innovations include the adaptive weight allocation mechanism that dynamically responds to changing signal quality, the asynchronous data alignment algorithm specifically calibrated for sport-specific movement patterns, and the multi-scale feature extraction approach that captures coordination phenomena across different temporal and spatial resolutions. These methodological advances collectively enable more comprehensive, accurate, and contextually relevant analysis of team collaborative dynamics than previously attainable through single-sensor or conventional fusion approaches.

The comprehensive collaborative dynamics indicator system developed in this research provides a structured framework for quantifying previously subjective aspects of team coordination. By establishing measurable parameters for spatial synchronization, temporal coordination, and collective adaptability, this research bridges the gap between theoretical sports science concepts and practical performance analysis. The demonstrated correlations between specific coordination metrics and performance outcomes validate the practical utility of this approach, while the ability to identify characteristic coordination signatures of different tactical systems enhances its application across diverse sporting contexts.

From a theoretical perspective, this research contributes to the understanding of team sports as complex dynamical systems by providing empirical methods to quantify emergent coordination properties. The identification of specific coordination mechanisms underlying successful team performance advances knowledge beyond descriptive models toward explanatory frameworks with predictive capacity. The integration of individual, dyadic, and team-level analyses within a unified methodological approach facilitates more nuanced understanding of how coordination emerges across multiple organizational scales within team structures.

The practical significance of this research is demonstrated through its successful implementation across multiple sporting contexts and performance levels. The system’s capacity to provide actionable insights for tactical analysis, training intervention design, and performance evaluation addresses long-standing challenges in translating advanced measurement technology into practical team sport applications. The positive user feedback from coaches, analysts, and sport scientists validates the system’s utility beyond academic interest, suggesting potential for widespread adoption in professional and developmental sporting environments.

Despite these advances, several limitations must be acknowledged. The current implementation faces technical constraints including battery life limitations that restrict continuous monitoring during extended competitions, computational demands that necessitate dedicated processing resources for real-time analysis, and user interface complexity that creates adoption barriers for non-technical practitioners. Additionally, while the system demonstrates cross-sport applicability, sport-specific customization requirements still impose implementation costs that may limit accessibility for resource-constrained environments.

Future research directions should address these limitations through several approaches. Technical advancement should focus on edge computing optimization to reduce transmission bandwidth requirements and extend battery life, while machine learning enhancements could improve pattern recognition capabilities without increasing computational demands. Methodological refinements should explore deeper integration of perceptual-cognitive factors into the coordination analysis framework, potentially through incorporation of gaze tracking and decision-making measures. Application expansion should investigate longitudinal coordination development across athlete maturation to establish age-appropriate reference values and developmental trajectories.

The fusion methodology and analytical framework developed in this research have potential applications beyond sport contexts. The principles of multi-sensor integration for collaborative dynamics analysis could be adapted for military unit coordination assessment, emergency response team evaluation, performing arts ensemble synchronization, and industrial workplace safety monitoring. Each application domain would require specific contextual adaptation but could benefit from the fundamental mathematical framework for quantifying collective behavior patterns across multiple independent agents operating toward shared objectives.

In conclusion, this research establishes a robust methodological foundation for applying advanced sensor technology to the analysis of collaborative dynamics in team sports. By addressing the technical challenges of heterogeneous data integration and the conceptual challenges of quantifying complex coordination phenomena, this work contributes both practical analytical tools and theoretical frameworks that advance understanding of how effective team coordination emerges, develops, and translates into performance outcomes. Specifically, our approach overcomes three critical limitations of existing methods: (1) real-time processing constraints through edge computing optimization and adaptive sampling, (2) sensor reliability issues via dynamic weight allocation and redundant data streams, and (3) practical applicability gaps through user-centered design and validated coaching integration protocols. The demonstrated 73.6% prediction accuracy for match outcomes using coordination metrics represents a substantial advancement over traditional performance indicators (52–59% accuracy).

Future refinements will further enhance accessibility and application scope, potentially transforming how team dynamics are understood, measured, and developed across sporting and non-sporting domains.

## Data Availability

The datasets used and/or analysed during the current study available from the corresponding author on reasonable request.
